# Neonatal amygdala microstructure and structural connectivity are associated with autistic traits at 2 years of age

**DOI:** 10.1016/j.dcn.2026.101721

**Published:** 2026-04-07

**Authors:** Kadi Vaher, Samuel R. Neal, Manuel Blesa Cábez, Lorena Jiménez-Sánchez, Amy Corrigan, David Q. Stoye, Helen L. Turner, Rebekah Smikle, Hilary Cruickshank, Magda Rudnicka, Mark E. Bastin, Michael J. Thrippleton, Rebecca M. Reynolds, James P. Boardman

**Affiliations:** a Centre for Reproductive Health, Institute for Regeneration and Repair, The University of Edinburgh, Edinburgh, UK; b Centre for Clinical Brain Sciences, The University of Edinburgh, Edinburgh, UK; c Salvesen Mindroom Research Centre, The University of Edinburgh, Edinburgh, UK; d Simpson Centre for Reproductive Health, Royal Infirmary Edinburgh, Edinburgh, United Kingdom; e Centre for Cardiovascular Science, University of Edinburgh, Edinburgh, UK

**Keywords:** Amygdala, Diffusion MRI, Q-CHAT, Autistic traits, Network-based statistics, Cortisol

## Abstract

Prenatal maternal stress is linked to neurodevelopmental outcomes. Maternal hair cortisol concentration in pregnancy is associated with neonatal amygdala microstructure and structural connectivity, suggesting that amygdala is sensitive to antenatal stress. We investigated whether amygdala microstructure and/or connectivity associate with neurodevelopmental outcomes. 174 participants (105 preterm) underwent brain MRI at term-equivalent age and assessment of neurodevelopment, autistic traits, temperament, and executive function at 2 years corrected age. We calculated amygdala microstructure (fractional anisotropy, mean diffusivity, neurite density index, orientation dispersion index) and structural connectivity (mean fractional anisotropy) to 6 regions (insula, putamen, thalamus, inferior temporal gyrus, medial orbitofrontal cortex, rostral anterior cingulate cortex). We used linear regression to model amygdala-outcome associations, adjusting for gestational age at birth and at scan, sex, maternal education and postnatal depression score, and network-based statistics (NBS) for whole-brain analyses. Following correction for multiple comparisons, lower amygdala mean diffusivity (left: β = -0.32, p = 0.026, right: β = -0.38, p = 0.012), higher left amygdala neurite density index (β = 0.35, p = 0.026), and increased left amygdala-putamen connectivity (β = 0.31, p = 0.026) associated with higher autistic traits across the whole sample. NBS additionally revealed amygdala-involving networks associated with cognition and surgency among preterms, and gestation-dependent associations with autistic traits. Findings indicate that neonatal amygdala microstructure may be important in the development of autistic traits.

## Highlights

• Amygdala development is sensitive to the impact of antenatal stress.
• We integrate neonatal brain MRI and 2-year developmental outcome data (n = 174).
• We use regression models and network-based statistics (NBS).
• Amygdala microstructure and structural connectivity associate with autistic traits.
• NBS showed amygdala networks in relation to cognition and surgency in preterms.

## Graphical Abstract

**Figure:**
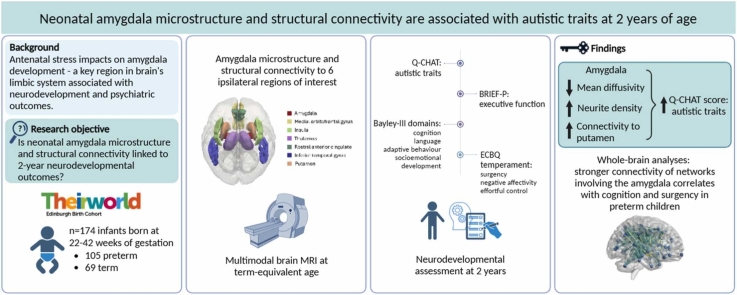

## 1 Introduction

Exposure to prenatal stress, commonly experienced by women during pregnancy (Woods et al., 2010), is associated with cognitive, behavioural and mental health conditions across the life course, including higher rates of anxiety and depression, negative affectivity, autistic traits, and diagnoses such as attention deficit hyperactivity disorder and schizophrenia (Collins et al., 2024, Van den Bergh et al., 2020), often in a sexually dimorphic manner (Sutherland and Brunwasser, 2018). Dysregulation of the hypothalamic-pituitary-adrenal (HPA) axis may be the principle mechanism whereby maternal stress influences offspring brain development (Moisiadis and Matthews, 2014). The amygdala is central to emotional and social information processing (LeDoux, 2007), and is a compelling target for neurodevelopmental programming via the HPA axis. The amygdala develops early in gestation (Humphrey, 1968), has a high concentration of glucocorticoid receptors (Wang et al., 2014), and undergoes protracted development into adolescence (Schumann et al., 2004, Zhou et al., 2021).

Magnetic resonance imaging (MRI) studies have revealed that exposure to prenatal stress is associated with alterations in structural and functional connectivity in brain networks involving the amygdala (Franke et al., 2020, Scheinost et al., 2016). Specifically, we previously showed that maternal hair cortisol concentration sampled soon after birth—a marker of cortisol levels over the last 3 months of pregnancy—was associated with neonatal amygdala microstructure and structural connectivity in a sexually dimorphic manner (Stoye et al., 2020). These findings are aligned with earlier work showing associations between maternal salivary cortisol levels during pregnancy and functional connectivity of amygdala-involving networks in neonates (Graham et al., 2019) and amygdala volumes in childhood (Buss et al., 2012). Furthermore, self-reported/perceived maternal prenatal stress correlates with amygdala volume (Acosta et al., 2019), grey matter microstructure (Rifkin-Graboi et al., 2013), and structural (Posner et al., 2016) and functional connectivity (Posner et al., 2016, Qiu et al., 2015, Soe et al., 2018).

Neuroimaging studies have also demonstrated associations between amygdala anatomy and numerous neurodevelopmental and psychiatric conditions such as autism spectrum disorder, anxiety and schizophrenia (Schumann et al., 2011). Methodological advances in MRI techniques over the past decade have further enabled characterisation of the neonatal amygdala in relation to childhood outcomes. Increased amygdala volume at term-equivalent age has been associated with poorer working memory (Nolvi et al., 2021) and heightened fear response in toddlers (Cismaru et al., 2016). Furthermore, altered resting-state functional connectivity of the neonatal amygdala to other brain regions correlates with fear and sadness (Graham et al., 2016, Thomas et al., 2019), increased internalising behaviour (Graham et al., 2019, Rogers et al., 2017), atypical social communication (Scheinost et al., 2022), and socio-emotional development in childhood (Kanel et al., 2022). Some amygdala-behaviour associations may be sexually dimorphic although evidence is inconsistent (Graham et al., 2019, Hay et al., 2020, Nolvi et al., 2021).

Diffusion MRI (dMRI) provides a powerful tool to probe the microstructural properties of the developing brain, which may be particularly sensitive to the effects of prenatal stress on the developing amygdala, as we have previously shown (Stoye et al., 2020). Conventional diffusion tensor imaging (DTI) metrics, such as fractional anisotropy (FA) and mean diffusivity (MD), reflect different brain tissue properties, including neuronal density, fibre orientation dispersion, degree of myelination, free-water content and axonal diameter, but they are non-specific (Boardman and Counsell, 2019). Approaches to analysing dMRI data using biophysical models such as neurite orientation dispersion and density imaging (NODDI) aim to disentangle these different factors by separating the influence of neurite density and orientation dispersion from each other, yielding indices of orientation dispersion index (ODI), which captures the degree of dispersion of axonal fibre orientations (e.g. through fanning, bending, crossing) or dendrite orientations, and neurite density index (NDI), represented by the intracellular volume fraction (Boardman and Counsell, 2019, Zhang et al., 2012). DTI and NODDI have provided key insights into the microstructural organisation of the developing brain (Boardman and Counsell, 2019, Niu et al., 2025, Pecheva et al., 2018) and such measures could capture properties of the neonatal amygdala that may be relevant to later cognitive and behavioural outcomes.

The literature to date on amygdala-outcomes relationships is limited by relatively small sample sizes (median n = 58) and a paucity of studies including infants born very preterm, and yet people born preterm are disproportionally affected by cognitive and socio-emotional difficulties and have a higher prevalence of neurodevelopmental and psychiatric diagnoses (Johnson and Marlow, 2017, Wolke et al., 2019), and stress-amygdala interactions are expected to operate across the whole of gestation. Importantly, current literature leaves uncertainty about the associations between neonatal amygdala microstructure or structural connectivity probed from dMRI – features we previously demonstrated to associate with prenatal maternal cortisol levels (Stoye et al., 2020) – and subsequent neurodevelopment.

To address these knowledge gaps, we investigated relationships between neonatal amygdala microstructure and structural connectivity, and a range of neurodevelopmental and behavioural outcomes at two years of age in a sample enriched for prematurity.

## 2 Methods and materials

### 2.1 Participants

Participants were very preterm (gestational age (GA) at birth ≤ 32 completed weeks) and term-born infants recruited to the prospective longitudinal Theirworld Edinburgh Birth Cohort (TEBC) study (Boardman et al., 2020). Infants were born and recruited at the Royal Infirmary of Edinburgh, UK, between September 2016 and September 2021. The study was conducted according to the principles of the Declaration of Helsinki, and ethical approval was obtained from the UK National Research Ethics Service (South East Scotland Research Ethic Committee 16/SS/0154). Parents provided written informed consent.

Exclusion criteria were congenital malformation, chromosomal abnormality, cystic periventricular leukomalacia, haemorrhagic parenchymal infarction, and post-haemorrhagic ventricular dilatation. These criteria mean the cohort is representative of most survivors of modern intensive care practices (Boardman et al., 2020). The conventional neonatal MRI results from the cohort have been reported previously (Sullivan et al., 2024).

Here, we included participants who had data available for multimodal brain MRI acquired at term-equivalent age and at least one outcome measure at 2 years of corrected age.

### 2.2 Demographic and clinical information

Participant demographic and clinical information was collected through questionnaires and medical records. Please see Table S1 for details regarding the coding and definitions of the variables.

### 2.3 Magnetic resonance imaging

#### 2.3.1 Image acquisition

Infants were scanned at term-equivalent age in natural sleep at the Edinburgh Imaging Facility, Royal Infirmary of Edinburgh, University of Edinburgh, UK, using a Siemens MAGNETOM Prisma 3 T MRI clinical scanner (Siemens Healthcare Erlangen, Germany) as previously described (Blesa et al., 2021, Boardman et al., 2020, Galdi et al., 2024, Vaher et al., 2022). See Supplementary information for details.

#### 2.3.2 Image pre-processing

All raw T2w and dMRI images were inspected and any dataset with unusable data (e.g. excessive motion) was excluded from downstream processing. Image data were processed and networks constructed as previously described (Blesa et al., 2021, Galdi et al., 2024, Stoye et al., 2020, Vaher et al., 2022).

dMRI processing was performed as follows: for each subject, the two dMRI acquisitions were first concatenated and then denoised using a Marchenko-Pastur-PCA-based algorithm with MRtrix3’s command *dwidenoise* (Tournier et al., 2019, Veraart et al., 2016); eddy current, head movement and EPI geometric distortions were corrected using outlier replacement and slice-to-volume registration (Andersson et al., 2017, Andersson et al., 2016, Andersson et al., 2003, Andersson and Sotiropoulos, 2016) using *topup* and *eddy* implemented in FMRIB Software Library (FSL) (Smith et al., 2004); bias field inhomogeneity correction was performed by calculating the bias field of the mean b0 volume and applying the correction to all the volumes (Tustison et al., 2010) using MRtrix3’s *dwibiascorrect*.

The T2w images were processed using the minimal processing pipeline of the Developing Human Connectome Project (dHCP) (Makropoulos et al., 2018). This pipeline includes motion and bias-field correction, brain extraction, tissue segmentation, cortical surface reconstruction, and registration to a neonatal template. This allowed us to obtain the bias-corrected T2w images, brain masks, and the different tissue probability maps.

Finally, the mean b0 EPI volume of each subject was co-registered to their structural T2w volume using boundary-based registration (Greve and Fischl, 2009). T2-weighted images were used as the structural reference for co-registration, as they provide superior grey-white matter and tissue-CSF contrast in neonates compared to T1-weighted images.

Melbourne Children's Regional Infant Brain (M-CRIB) atlas parcellation (Alexander et al., 2017) was used to obtain regional parcellation. The 10 manually labelled subjects of the atlas were registered to the bias field corrected T2w using rigid, affine and symmetric normalisation (SyN) (Avants et al., 2008). The registered labels of the 10 atlases were then merged using joint label fusion (Wang et al., 2013), resulting in a parcellation containing 84 regions of interest (ROIs). All these steps were performed with *antsJointLabelFusion.sh*.

dMRI image quality was evaluated using eddy QC (Bastiani et al., 2019), which was performed at both individual-subject and group levels. Eddy QC provides several metrics, including the mean absolute and relative motion, the signal-to-noise ratio (SNR) of the b0 volumes, and the contrast-to-noise ratio (CNR) maps for each b-value. For eddy QC to work, we excluded the b = 200 s/mm^2^ shell, because the low number of volumes with this b-value occasionally causes the Gaussian-process model used by eddy to produce unrealistically perfect fits, leading to implausible CNR maps. Overall, the dataset demonstrated good image quality (Figure S1), comparable to what we previously reported in the TEBC cohort (Galdi et al., 2020). We compared the distributions of the quality metrics between term and preterm infants using Wilcoxon rank-sum test and found no statistically significant differences in the average absolute (p = 0.239) or relative motion (p = 0.127) or SNR (p = 0.901), though there were some differences in the CNR at the different b-values (b 500: p = 0.001; b 750: p = 4.01 × 10^−9^; b 2500: p = 1.99 × 10^−9^). To further ensure accurate alignment, we visually inspected a subset of subjects to confirm proper registration between the dMRI and T2w images and no major misalignments were present. No data were removed following QC.

#### 2.3.3 Network construction and analysis

Tractography and fractional anisotropy-weighted connectomes were constructed as previously published (Stoye et al., 2020). We performed anatomically constrained tractography using constrained spherical deconvolution (Smith et al., 2012, Tournier et al., 2019). We used FA threshold of 0.1 to calculate the multi-tissue response function, followed by calculation of the average response functions. Then, multi-tissue fiber orientation distribution (FOD) was calculated (Jeurissen et al., 2014) and global intensity normalisation on the FODs images was performed. Finally, the tractogram was created, generating 10 million streamlines, with a minimum length of 20 mm and a maximum of 200 mm and a cut-off of 0.05 (default), using backtrack and a dynamic seeding (Smith et al., 2015a). To be able to quantitatively assess connectivity, spherical-deconvolution informed filtering of tractograms (SIFT2) was applied to the resulting tractograms (Smith et al., 2015a). The connectivity matrix was constructed using a robust approach, a 2 mm radial search at the end of the streamline was performed to allow the tracts to reach the grey matter parcellation (Smith et al., 2015b). The final connectivity matrices were multiplied by the *m* coefficient obtained during the SIFT2 process. These connectomes gave a quantification of the SIFT2 weights (referred to as the streamline counts), and the mean FA of connections, between both the left and right amygdala to 41 unilateral regions of interest defined through M-CRIB parcellation.

#### 2.3.4 Image feature extraction

**Microstructural features:** DTI and NODDI maps were calculated in the dMRI processed images to obtain FA, MD, NDI and ODI. The DTI model was fitted in each voxel using the weighted least-squares method DTIFIT as implemented in FSL (Jenkinson et al., 2012) using only the b = 750 s/mm^2^ shell. NODDI metrics were calculated using the original NODDI toolbox using all shells and the recommended values for neonatal grey matter of the parallel intrinsic diffusivity (1.25 μm^2^/ms) (Guerrero et al., 2019, Zhang et al., 2012). NODDI toolbox was also used to obtain the isotropic water fraction (ISO) maps. To minimise partial volume effects, we excluded voxels with ISO < 0.5 (Batalle et al., 2019) and calculated the mean FA, MD, ODI, and NDI for the left and right amygdalae from the Melbourne Children's Regional Infant Brain atlas parcellation (Alexander et al., 2017).

**Fig. 1:**
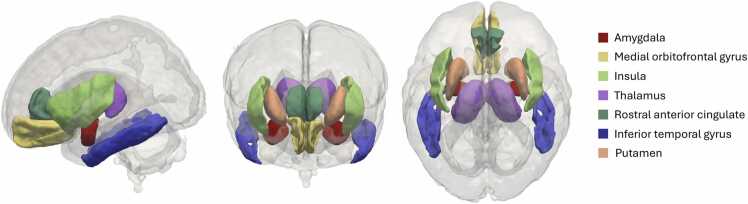
Segmentations of the amygdalae and connected regions of interest in the ENA50 neonatal atlas space. Shown in lateral (left), anterior (centre) and superior (right) views.

**Structural connectivity features:** We selected four connections of interest from the amygdalae, extracted separately for the left and right hemispheres, as predictors of neurodevelopmental outcomes due to their (nominally) significant associations with either the interaction effect between maternal hair cortisol concentration and infant sex, or with maternal hair cortisol concentration in sex-stratified analyses in our previous work (Stoye et al., 2020): putamen, thalamus, insula, and inferior temporal gyrus. We selected two additional connections from the amygdalae to the medial orbitofrontal cortex and rostral anterior cingulate cortex due to their associations with behavioural outcomes in other paediatric studies (Graham et al., 2016, Hay et al., 2020, Phillips et al., 2021, Thomas et al., 2019). Mean FA for each connection was calculated as an indicator of structural connectivity strength/integrity. Segmentation of the amygdalae and the 6 connected regions of interest are visualised in Fig. 1.

### 2.4 Follow-up appointment and behavioural testing

At 2 years of corrected age, we collected information about participants’ neurodevelopment. Primary caregivers were asked to complete questionnaires to assess neurodevelopment across domains.

**Early Childhood Behavioural Questionnaire (ECBQ):** ECBQ assesses temperament between the ages of 18 and 36 months (Putnam et al., 2006). From the questionnaire data, we calculated the three established factors that represent broad dimensions of temperament: Negative Affectivity, Surgency/Extraversion, and Effortful Control. Higher scores indicate higher prevalence of behaviours indicative of these traits.

**Behaviour Rating Inventory of Executive Function, Preschool Version (BRIEF-P)**: BRIEF-P assesses the range of behavioural manifestations of infant’s executive function (Gioia et al., 2003). We calculated the Global Executive Composite score, with higher scores indicative of poorer executive functioning. Infants with elevated ratings on either of the two validity indices of BRIEF-P, negativity and inconsistency (n = 4), were excluded from the analyses.

**Quantitative Checklist for Autism in Toddlers (Q-CHAT):** Q-CHAT assesses frequency of behaviours also observed in autism spectrum conditions in toddlers aged 18–30 months with the aim of identifying those who should be referred for diagnostic assessment (Allison et al., 2021). We obtained a total Q-CHAT score, with higher scores indicative of higher frequency of autistic traits. Missing questionnaire items were replaced with zeros to calculate the total Q-CHAT score, assuming at least one item was completed (Allison et al., 2008); there were 12 infants with a range of 1–3 questionnaire item values missing which were replaced with zeros to calculate the total score.

**Bayley Scales of Infant and Toddler Development, Third Edition (Bayley-III):** Preterm infants additionally completed the Bayley-III (Bayley, 2006) with a trained clinician at 2 years of corrected age (Bayley, 2006) as part of their routine National Health Service developmental follow-up appointment. From medical records, we obtained the standardised composite scores for four domains measured with Bayley-III: cognitive, language, adaptive, and social-emotional development, with higher scores indicative of better functioning in these domains. To reduce the number of tests, the motor domain was omitted as amygdala is not a dominant component of the motor system.

### 2.5 Statistical analysis

Statistical analyses were performed in R (version 4.3.2) (R Core Team, 2023).

#### 2.5.1 Covariates

Initially, the following covariates were included based on known associations with neonatal brain structure and/or neurodevelopment and their correlation with at least one outcome measure in the current sample: GA at birth, sex, ethnicity, maternal postnatal depression score (Edinburgh Postnatal Depression Scale (Cox et al., 1987), dichotomised as ≤ 10), maternal age and maternal final education qualification. We also adjusted for GA at scan due to its strong correlation with brain MRI metrics. See further details on covariate selection in Supplementary information.

Given prior evidence, we originally considered infant sex*MRI feature interaction term in the models. The interaction term was not consistently associated with the outcomes (p < 0.05 in 3/180 models) and model comparisons using analysis of variance (ANOVA), and Akaike and Bayesian information criteria did not indicate improvement in fit when including the interaction term, except for the 3 models. Therefore, the sex interaction effect was dropped.

Then, in the interest of constructing a parsimonious model, we removed infant ethnicity and maternal age because these variables associated with only one outcome. Full and sparser model comparisons revealed that the sparser model fit the data as well (not shown). Therefore, the final covariates included in analyses presented in the Results are: GA at birth, infant sex, GA at scan, maternal education, and postnatal depression score.

#### 2.5.2 Main analyses

Separate multiple linear regression models were constructed to test for associations between each neurodevelopmental outcome (dependent variable) and MRI feature (independent variable) for the left and right amygdala. In total, we tested 9 outcome scores and 10 MRI features for each hemisphere, resulting in 180 models.

**Fig. 2:**
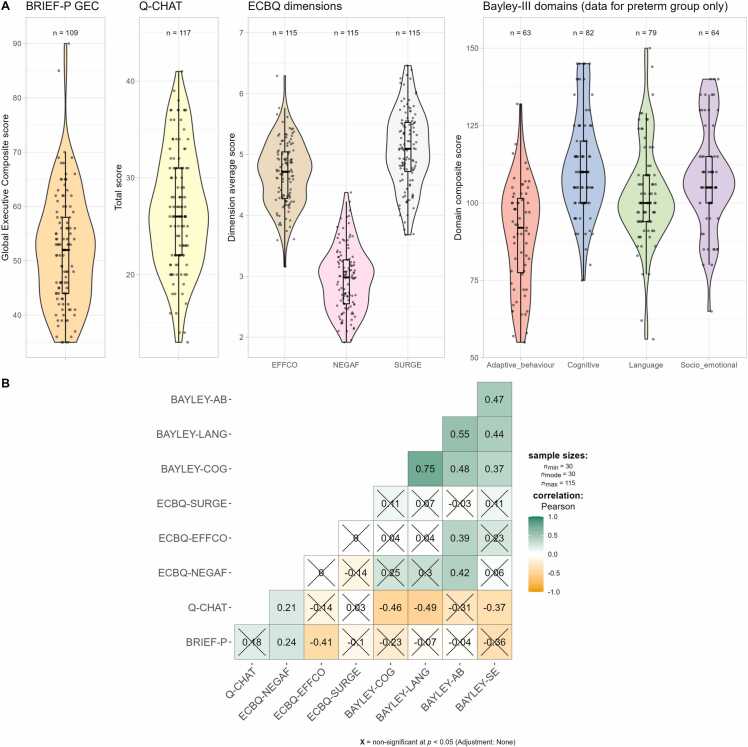
Distributions (A) and Pearson correlations (B) of 2-year outcome variables. Crossed out boxes indicate statistically non-significant correlations. BRIEF-P = Behavior Rating Inventory of Executive Function, Preschool, GEC = global executive composite, Q-CHAT = Quantitative Checklist for Autism in Toddlers; ECBQ = Early Childhood Behavior Questionnaire; EFFCO = effortful control, NEGAF = negative affectivity, SURGE = surgency, AB = adaptive behaviour, LANG = language, COG = cognitive, SE = socio-emotional.

To maximise sample size, analyses were performed on the subset of infants with available data for each outcome, resulting in nine subsets of our study sample (see Fig. 2 for sample sizes).

We tested model assumptions of linearity, homoscedasticity, and normality of residuals by visually inspecting the model diagnostic plots; presence of multicollinearity was determined by calculating the variance inflation factor for each covariate. An exemplary illustration of the model diagnostic plots is provided as Figure S2.

All continuous variables were z-transformed (mean-centered and scaled to unit variance) prior to fitting the models to enable comparability of effect sizes across predictors and allow interpretation of the results as standardised coefficients (i.e., change in outcome per 1 SD change in the predictor). Thus, we report standardised β coefficients and 95% confidence intervals (CI). We controlled the false discovery rate (FDR) at 0.05 using the Benjamini-Hochberg (Benjamini and Hochberg, 1995) procedure applied separately to each outcome measure. Results with corrected p-value < 0.05 were considered statistically significant.

#### 2.5.3 Subgroup analyses

Given that Bayley-III data were only available for preterm infants, we performed subgroup analyses separately within the term and preterm groups following the same model specifications to investigate gestation-dependent associations. As above, these models were also adjusted for FDR using the Benjamini-Hochberg procedure applied separately to each outcome measure within each group.

#### 2.5.4 Sensitivity analyses

Three sensitivity analyses were carried out.

First, because the variation in amygdala microstructure and its connectivity to other brain regions may be transmitting maternal factors to infant outcomes, we repeated all main models excluding maternal factors (education, postnatal depression score). The full model and the one without maternal factors were compared using ANOVA, Akaike and Bayesian information criteria, the adjusted R^2^, and the standardised β coefficients of the amygdala features. These models were corrected for multiple comparisons in the same manner as the main models.

Second, this dataset contained 14 twin pairs (13 preterm). We re-ran the four statistically significant main models, excluding one randomly selected twin from each pair. The four models were also adjusted for multiple comparisons using the Benjamini-Hochberg procedure.

Third, to explore the robustness of the amygdala-outcome associations, we repeated the four models with significant amygdala-outcome associations in the sample of our previous work showing associations between maternal hair cortisol and amygdala microstructure/connectivity (Stoye et al., 2020). Out of the n = 78 in the previous study, 39 (8 preterm [20.5%]) had data available for Q-CHAT and were included in this sensitivity analysis. No correction for multiple comparisons was performed here.

#### 2.5.5 Network based statistics

We performed network-based statistics (NBS) (Zalesky et al., 2010) to identify whether the amygdala might be part of a larger network that is associated with outcomes. Edge-wise comparison of connectivity matrices was performed using *connectomestats* within MRtrix3 (Tournier et al., 2019). NBS results are highly dependent on the primary test-statistics threshold. Therefore, given the lack of an established test-statistic threshold to use in NBS analysis of neonatal/paediatric data, we tested a range of values (t = 1.5–3.5, with 0.1 increments; i.e. 21 experiments) (Ní Bhroin et al., 2020). All other parameters were set to their default values. The NBS analyses, similarly to main regression models, were adjusted for GA at birth, infant sex, GA at scan, maternal education, and postnatal depression score; continuous variables were z-transformed. *Connectomestats* for NBS analyses internally corrects for multiple testing using permutation testing and family-wise error rate control with threshold-free cluster enhancement; as with regression models, all multiple comparison adjustments were conducted per outcome measure.

We took a multi-verse approach, reasoning that confidence in the implicated connections is increased if they appear under several test-statistic values. Therefore, focussing on results for which a connection with amygdala was part of the statistically significant outcome-associated network (p < 0.05), for each connection we calculated the proportion of experiments where it was part of the associated network. Then, we filtered this to focus on and visualise only those connections that appeared in at least 7/21 (33.3%) of experiments, considering them as consistent and less dependent on the selected statistic threshold. Similarly to regression models, we additionally ran subgroup analyses in term and preterm groups separately.

### 2.6 Data and code availability

Data used for analysis in this work are deposited in Edinburgh DataVault (Boardman et al., 2022) (https://doi.org/10.7488/e65499db-2263–4d3c-9335–55ae6d49af2b). Requests for access for these as well as raw neuroimaging data will be considered under the study's Data Access and Collaboration policy and governance process (https://www.ed.ac.uk/centre-reproductive-health/tebc/about-tebc/for-researchers/data-access-collaboration, James.Boardman@ed.ac.uk). Code used for the data analysis in this paper is available on GitLab (https://git.ecdf.ed.ac.uk/jbrl/amy-neurodev).

## 3 Results

### 3.1 Participant characteristics

174 participants (characteristics shown in Table 1) had available data for multimodal neonatal brain MRI and at least one outcome measure collected at 2 years of life. The distributions and intercorrelations of the 2-year outcome variables along with sample sizes available for each are presented in Fig. 2. Q-CHAT scores were higher in preterm compared to term infants (t = –4.345, p < 0.001), though only two preterm participants had scores > 39, a screening cut-off point shown to maximise sensitivity and specificity for autism diagnosis (Allison et al., 2021). In addition, ECBQ negative affectivity trait was slightly lower in the term group (t = –1.901, p = 0.060; Figure S3). Figure S4 shows that the distribution of responses for individual Q-CHAT items were broadly similar across the two groups, with the preterm group showing slightly higher mean values for most items (based on visual inspection; item-level scores were not statistically tested between term and preterm groups). Figure S5 shows the distribution of the neonatal amygdala MRI metrics in term and preterm groups. After adjusting for multiple comparisons, linear regression models adjusting for GA at MRI revealed that preterm infants had statistically significantly lower FA and higher ODI values in the amygdala bilaterally, while all structural connectivity values, except between right amygdala and right insula, were significantly lower in the preterm compared to the term group (Table S2).

**Table 1:** Summary of infant and maternal demographics.

Characteristic*	Overall n = 174	Term n = 69	Preterm n = 105	p-value#
**Infant**				
Male	103 (59%)	39 (57%)	64 (61%)	0.700
Gestation at birth (weeks)	31.43 [22.14–42.14]	39.71 [36.43–42.14]	30.00 [22.14–32.29]	< 0.001
Birthweight (g)	1690 [370–4560]	3460 [2410−4560]	1330 [370−2510]	< 0.001
Birthweight *z*-score	0.20 (1.03)	0.44 (1.01)	0.05 (1.02)	0.015
Gestation at MRI (weeks)	41.20 (1.53)	42.11 (1.18)	40.59 (1.44)	< 0.001
**Maternal**				
Age at booking (years)	33.4 (5.0)	34.6 (4.2)	32.7 (5.4)	0.011
BMI at booking (kg/m^2^)	25.1 [16.4–47.0]	24.6 [18.2–47.0]	25.5 [16.4–46.6]	0.059
University or postgraduate education	121 (70%)	60 (87%)	61 (58%)	< 0.001
Edinburgh Postnatal Depression Scale ≥ 10	42 (24%)	13 (19%)	29 (28%)	0.300
**Outcome measures**				
BRIEF-P GEC	52 (10)	50 (8)	53 (12)	0.11
Q-CHAT	26.7 (6.1)	24.6 (5.8)	29.1 (5.5)	< 0.001
ECBQ EFFCO	4.68 (0.53)	4.65 (0.49)	4.71 (0.57)	0.5
ECBQ NEGAF	2.96 (0.52)	2.88 (0.49)	3.06 (0.55)	0.060
ECBQ SURGE	5.08 (0.63)	5.13 (0.62)	5.03 (0.63)	0.4
BAYLEY-COG			112 (16)	
BAYLEY-MOT			109 (12)	
BAYLEY-LANG			103 (16)	
BAYLEY-AB			90 (17)	
BAYLEY-SE			108 (17)	

BRIEF-P = Behavior Rating Inventory of Executive Function, Preschool, GEC = global executive composite, Q-CHAT = Quantitative Checklist for Autism in Toddlers; ECBQ = Early Childhood Behavior Questionnaire; EFFCO = effortful control, NEGAF = negative affectivity, SURGE = surgency, AB = adaptive behaviour, LANG = language, COG = cognitive, SE = socio-emotional.

* Data are presented as *n* (%) for categorical variables, mean (SD) for continuous and normally distributed variables, or median [range] for continuous non-normally distributed variables.

# Values in term and preterm group are compared with Chi-squared tests for categorical variables, *t*-tests for continuous and normally distributed variables, or Mann-Whitney U-test for continuous non-normally distributed variables.

### 3.2 Main analyses: amygdala microstructure and structural connectivity associate with autistic traits

**Fig. 3:**
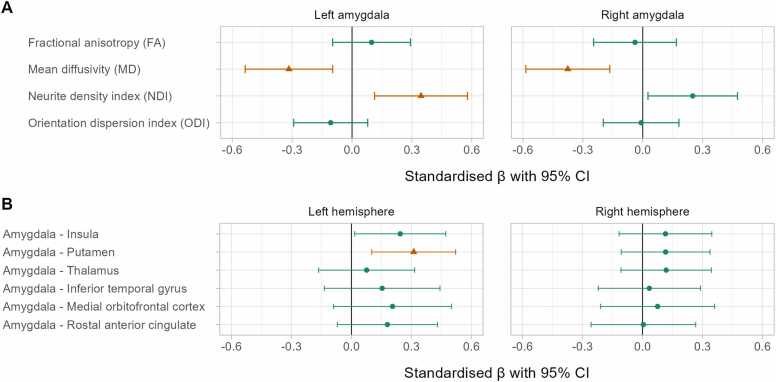
Amygdala microstructure (A) and structural connectivity (B) associations with Q-CHAT scores. Models are adjusted for gestational age at birth and at scan, infant sex, maternal education (university/postgraduate degree vs lower) and high postnatal depression screening score (Edinburgh Postnatal Depression Scale ≥ 10). Coefficients shown as orange lines and triangular shapes indicate statistically significant associations after adjustment for multiple comparisons using the Benjamini-Hochberg procedure.

After adjustment for multiple tests, amygdala microstructural indices showed statistically significant associations only with autistic traits (Fig. 3A): MD in the amygdala bilaterally was negatively (left: β = −0.316, 95% CI [−0.535 to −0.097]; right: β = −0.375, 95% CI [−0.586 to −0.165]) and NDI in the left amygdala positively (β = 0.346, 95% CI [0.113 to 0.579]) associated with Q-CHAT scores. The structural connectivity (mean FA) between left amygdala and putamen positively associated with Q-CHAT scores (β = 0.311, 95% CI [0.102 to 0.521]; Fig. 3B). We found no statistically significant associations between amygdala features and temperament (ECBQ), executive function (BRIEF-P) or Bayley-III composite scores (Table S3).

### 3.3 Sensitivity analyses

**Fig. 4:**
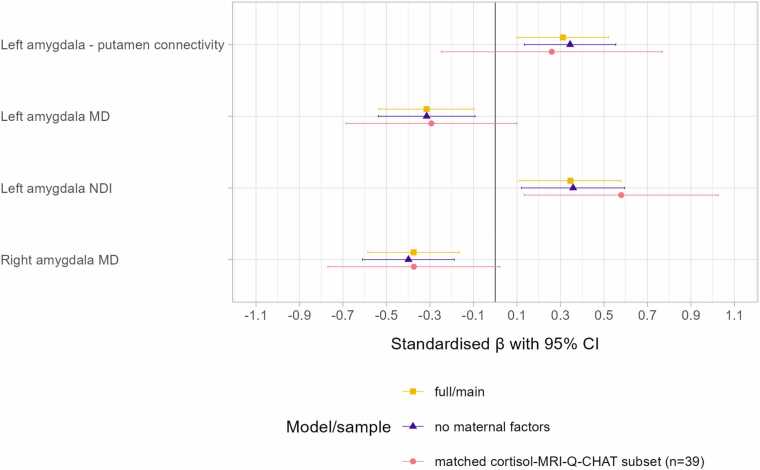
Amygdala microstructure and structural connectivity associations with Q-CHAT in sensitivity analyses in comparison with the main model. Full/main indicates the original model associating amygdala features with Q-CHAT adjusting for gestational age at birth, infant sex, gestational age at scan, maternal final education qualification, and risk for postnatal depression; no maternal factors indicates the original model but excluding maternal final education qualification and risk for postnatal depression; matched cortisol-MRI-Q-CHAT subset indicates the original model in a smaller subset who had data available for MRI, Q-CHAT, and prenatal maternal cortisol.

The association strength between amygdala microstructure/connectivity and Q-CHAT score was minimally altered when excluding maternal factors from the model (average change in standardised β of the imaging predictor: 0.018; Fig. 4), indicating that amygdala MRI features are associated with autistic traits independent of maternal education and postnatal depression score.

The four amygdala microstructure/connectivity and Q-CHAT associations remained statistically significant when excluding one twin randomly from the sample (n = 108), with little change to the regression coefficients (Table S4).

We repeated the significant amygdala-Q-CHAT models in a smaller subset of participants overlapping with our previous publication (Stoye et al., 2020) who had data available for cortisol, MRI and Q-CHAT. The standardised β-s were similar and within the 95% confidence intervals of the larger sample (Fig. 4). This consistency suggests that the observed amygdala-outcome associations are robust and not dependent on sample size, providing additional confidence in the reliability of these findings.

### 3.4 Subgroup analysis

The Q-CHAT negative associations with amygdala MD were similar in term and preterm group, while the positive associations with NDI and left amygdala-putamen connectivity were stronger in the term group (Table S5; Figure S6). In the term group, 2-year Q-CHAT scores were further correlated with left amygdala ODI as well as positively with several other amygdala connections, especially in the left hemisphere (Figure S6B). Interestingly, the structural connectivity between the amygdala and thalamus in the left hemisphere appears to have associations in opposite directions in term and preterm groups with a positive association observed among the term and a negative association among the preterm group. The majority of the associations remained statistically significant following adjustment for multiple comparisons in the term but not the preterm group.

### 3.5 Network-based statistics

**Fig. 5:**
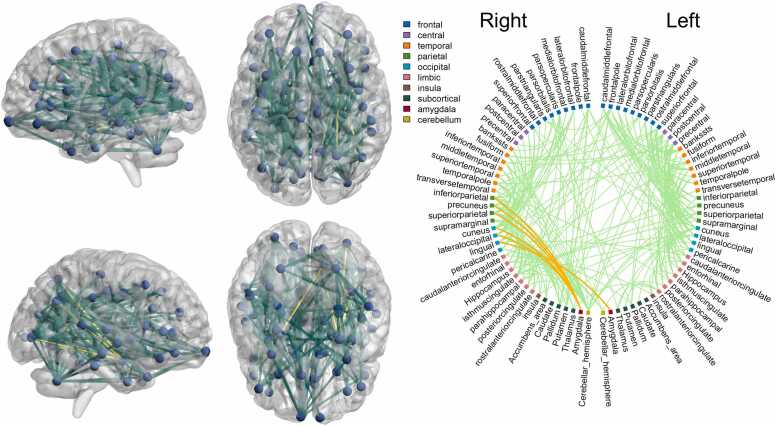
Illustration of network-based statistics results for Bayley-III cognitive domain. Connections that appear as significant in at least 33.3% of experiments are shown; amygdala connections (right amygdala to right cuneus, right superior parietal cortex, right inferior parietal cortex, right lateral occipital cortex and right precuneus, and left amygdala to right lingual cortex) are shown in yellow/orange. All connections within the visualised network were positively associated with cognitive scores; no significant negatively associated (sub)networks were identified.

Given the largely null fundings in our main analyses for amygdala structural connectivity, we used NBS to determine if amygdala is part of larger brain networks correlated with outcomes. We found five connections from the right amygdala to regions in the parietal and occipital lobes (right cuneus, right superior parietal cortex, right inferior parietal cortex, right lateral occipital cortex and right precuneus) and one connection from the left amygdala (to right lingual cortex) that were part of a brain-wide network which positively correlated with Bayley-III cognitive scores among preterm infants (Fig. 5). These six connections were part of the associated network at test-statistic thresholds from 1.5 to 2.1. Amygdala connections were not part of consistently associated networks for other 2-year outcome measures across the entire sample.

**Fig. 6:**
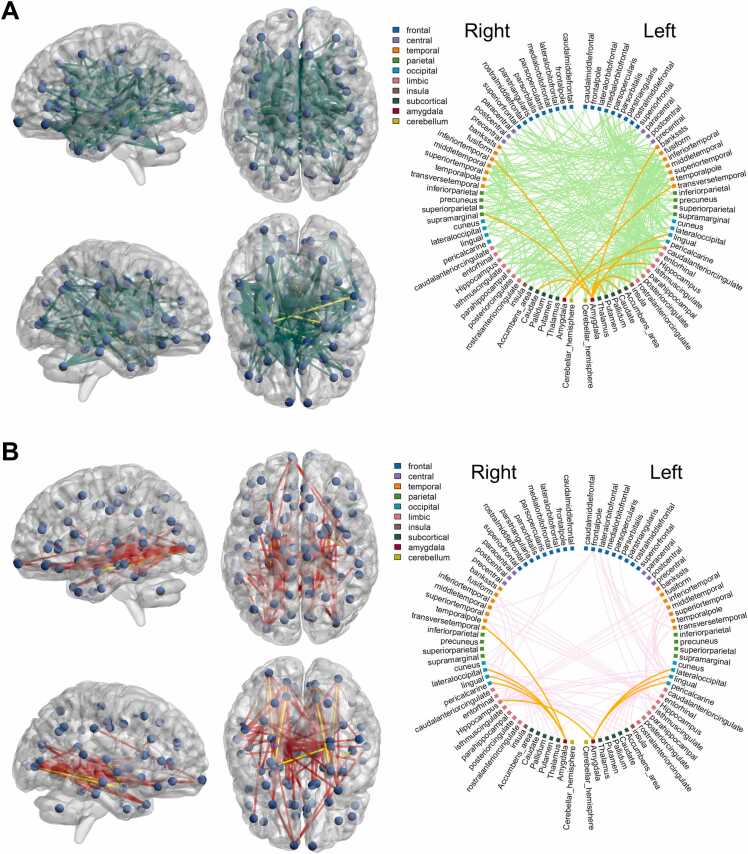
Illustration of network-based statistics results for Q-CHAT in term (A) and preterm (B) groups. Connections that appear as significant in at least 33.3% of experiments are shown; amygdala connections are shown in yellow/orange. In the term group (A), all connections were positively, while in the preterm group (B), all connections were negatively correlated with Q-CHAT scores. In the term groups the amygdala connections are: from the right amygdala to left caudal anterior cingulate, left isthmus cingulate and left transverse temporal gyrus, and from the left amygdala to left banks of the superior temporal sulcus, left lingual cortex, left insula, left caudate, left putamen, right caudate, right inferior temporal gyrus and right supramarginal gyrus. In the preterm group, the amygdala connections are: from the left amygdala to left lateral occipital cortex, left lingual cortex, left pericalcarine cortex and the right hippocampus, and from the right amygdala to the right lateral occipital cortex, right lingual cortex, right pericalcarine cortex and right transverse temporal cortex.

In subgroup analyses, with regards to Q-CHAT, among the term group, there were 3 connections from the right amygdala (to left caudal anterior cingulate, left isthmus cingulate and left transverse temporal gyrus) and 8 connections from the left amygdala (to left banks of the superior temporal sulcus, left lingual cortex, left insula, left caudate, left putamen, right caudate, right inferior temporal gyrus and right supramarginal gyrus) that were consistently, mostly at test-statistic thresholds from 1.5 to 2.3, part of a network whose connectivity positively correlated with Q-CHAT scores (Fig. 6A). In contrast, among the preterm group, NBS identified that amygdala was part of a network whose connectivity negatively correlated with Q-CHAT scores. The consistent connections, mostly at test-statistic thresholds from 2.1 to 2.9, included 4 connections from the left and right amygdalae primarily to regions in the occipital lobe (from the left amygdala to left lateral occipital cortex, left lingual cortex, left pericalcarine cortex and the right hippocampus, and from the right amygdala to the right lateral occipital cortex, right lingual cortex, right pericalcarine cortex and right transverse temporal cortex; Fig. 6B).

Subgroup analyses further revealed that the amygdala is part of a network whose connectivity is positively correlated with ECBQ surgency dimension in preterm infants (Figure S7). The amygdala connections include both inter- and intra-hemispheric connections, mostly with central and parietal cortical regions.

### 3.6 Maternal hair cortisol and Q-CHAT

Finally, we explored to what extent maternal hair cortisol concentration correlates with Q-CHAT scores (please see Supplementary information for details on cortisol measurements). There was no correlation between maternal hair cortisol concentration and Q-CHAT score across the entire sample (Figure S8A). However, there may be sex-specific relationships between maternal cortisol and Q-CHAT such that there is a negative correlation in male and positive correlation in female infants (Figure S8B; β_interaction_ = −0.661, p = 0.043, adjusted for GA at birth, n = 39).

## 4 Discussion

By combining multimodal brain MRI and developmental outcome data we investigated relationships between neonatal amygdala and a wide range of behavioural outcomes. Our study shows that neonatal amygdala microstructure and structural connectivity to putamen – previously linked to maternal cortisol levels in pregnancy – associate with autistic traits at 2 years of age. Whole-brain analyses further revealed an amygdala-involving network associated with cognitive scores in preterm infants and suggestions for gestation-dependent associations between brain connectivity and autistic traits.

Previous work has shown amygdala enlargement in autistic children, with positive correlations between amygdala volume and autistic traits (Mosconi et al., 2009, Nordahl et al., 2012, Schumann et al., 2011, Schumann et al., 2009), followed by reduced growth later, resulting in amygdala that is smaller than or of equal size to typically developing individuals in adolescence and adulthood (Schumann et al., 2011, Schumann et al., 2004). Histologic analyses have further found initial excess of amygdala neurons/neurites in autistic children (Avino et al., 2018). Our findings of higher NDI and lower MD in association with higher autistic traits appear consistent with this as these microstructural changes may indicate higher tissue/neurite and lower water content in the region.

Prior studies have further demonstrated atypical patterns of amygdala *functional* connectivity in autistic children, particularly with sensory regions (Fishman et al., 2018, Lee et al., 2020). More recently, altered functional connectivity between the amygdala and visual regions was reported in 12-month-old infants with higher genetic liability for autism (Liu et al., 2024). However, to our knowledge, associations between amygdala *structural* connectivity and autistic traits have not been explored. We found that stronger neonatal amygdala-putamen connectivity in the left hemisphere may be positively correlated with autistic traits at 2 years of age. Relatedly, prior work has implicated the basal ganglia and striatum, including the putamen, in autism, with differences in both anatomy and functional connectivity of these regions associated with autistic behaviours, diagnosis, or genetic risk scores (Di Martino et al., 2011, Estes et al., 2011, Wang et al., 2021). Intriguingly, altered functional connectivity between the amygdala and regions involved in social communication and repetitive behaviours, including the striatum, has also been reported in autistic 3-year-old boys (Shen et al., 2016). While there are some parallels in previous literature of amygdala and putamen involvement in autism, the associations found in the current study should not be over-interpreted because our study was not designed to specifically investigate autism; rather, Q-CHAT scores capture a continuum of socio-emotional, sensory and repetitive behaviours that may be relevant to autism (Allison et al., 2008). Notably, a recent fMRI study in a combined term and preterm sample found that dynamic features of specific neonatal brain states, in which putamen was a significant contributor, associated with Q-CHAT scores in toddlerhood (França et al., 2024). Collectively, these findings suggest a role for early amygdala–striatal circuitry in later socio-emotional and behavioural variation.

Our term/preterm segregated analyses suggest that there are gestation-dependent associations between amygdala features and autistic traits. Specifically, although our previous work showed similar cortisol–amygdala associations across term and preterm groups (Stoye et al., 2020), here, targeted regression analyses indicated stronger positive associations between structural connectivity in the selected amygdala pathways and autistic traits in term than in preterm group. Moreover, whole-brain NBS analyses revealed that the structural networks involving the amygdala may be associated with autistic traits in opposite direction of effect in the two groups: in term infants, higher structural connectivity of the neonatal amygdala was associated with a higher frequency of autistic traits in toddlerhood, whereas in preterm infants it was associated with a lower frequency. An example from the targeted regressions is the left amygdala-thalamus connection, which correlated positively with autistic traits in the term but negatively in the preterm group; however, this connection was not highlighted by NBS. Furthermore, NBS analyses revealed distinct amygdala connections to be involved in the Q-CHAT-associated networks in the term and preterm groups, including several additional amygdala connections beyond those associated with maternal hair cortisol levels in our previous study (Stoye et al., 2020). In preterm infants the network included amygdala connections primarily with occipital regions, while in term infants there were several connections with subcortical and limbic regions, and the insular cortex. Notably, the amygdala-insula connectivity showed consistent associations with autistic traits across both targeted regression and NBS analyses among the term group. This is particularly interesting given prior findings that lower functional connectivity between left amygdala and insula in neonates is correlated with family history of autism as well as higher social risk scores at 1 year of age (Scheinost et al., 2022). The groupwise differences in amygdala structural connectivity and Q-CHAT associations may be explained by differences in the neural basis of autism among children born preterm, in whom the multitude of preterm-specific exposures, such as systemic inflammation and gut microbiome alterations, are associated with altered microstructural properties and connectivity of the brain as well as autism (Boardman and Counsell, 2019, Bokobza et al., 2019, Joseph et al., 2017, Rogers et al., 2017, Vaher et al., 2026, Vaher et al., 2024). Furthermore, the network differences we observed may offer new insights into the heterogeneity of autism phenotypes and atypical social cognition among children born preterm (Bowers et al., 2015, Dean et al., 2021, Joseph et al., 2023, Zhang et al., 2025). In sum, it is plausible that preterm birth exposes a susceptibility to autism mediated through both shared and more widely distributed networks than those associated with autism in children born at term.

To link our previous findings of sexually dimorphic cortisol-amygdala microstructure associations (Stoye et al., 2020) with the main findings of the current study, in a smaller overlapping sample we found evidence for a sex-specific relationship between maternal hair cortisol levels and Q-CHAT scores such that there was a negative correlation in males and a positive correlation in females. This is in line with previous studies suggesting a sex interaction effect between maternal blood cortisol levels in pregnancy and autistic behaviours in childhood, with particularly prominent associations in males, though there is mixed evidence about the direction of this association (Andreasen et al., 2023, Ram et al., 2019). However, direct comparisons of the three studies are complicated given their methodological differences in assessing autistic behaviours and cortisol. Our analyses did not find evidence for a sex-by-amygdala interaction, suggesting that the extent to which the cortisol-associated neonatal amygdala MRI features correlate with subsequent autistic traits is similar in males and females. These results together raise the possibility of a sex-moderated mediation effect such that higher prenatal cortisol levels in males may lead to changes in amygdala that are subsequently linked with lower frequency of autistic traits, whilst in females higher prenatal cortisol associates with changes in amygdala which in turn relate to higher frequency of autistic traits. This is akin to a report by Graham et al. (Graham et al., 2019) who found that neonatal amygdala connectivity was similarly correlated with internalising behaviours in male and female infants, but that amygdala connectivity mediated maternal cortisol-behaviour associations in females only. This interpretation should be approached with caution because the analyses discussed here stem from only partially overlapping samples. Therefore, future larger studies are encouraged to formally test this hypothesis of a sex-moderated mediation effect of the neonatal amygdala for autistic traits.

Whole brain connectivity analyses revealed increased neonatal structural connectivity of brain networks in a positive association with surgency trait and Bayley-III cognitive scores at 2 years of age in preterm infants. Amygdala was part of these networks, but not the sole contributor, and the 6 cortisol-associated connections of interest were not included in these networks. Increased amygdala functional connectivity in infancy has been mainly associated with higher negative affectivity traits in childhood, including fear and sadness (Graham et al., 2016, Thomas et al., 2019), internalising behaviours (Graham et al., 2019, Rogers et al., 2017), and lower positive affectivity (Filippi et al., 2021). In contrast, in our previous study, neonatal amygdala structure or whole brain structural connectivity did not associate with attachment behaviours at 9 months of age (Jiménez-Sánchez et al., 2024), though it is salient that the correlations between temperament traits and attachment are weak (Groh et al., 2017). Therefore, our results of a positive correlation with surgency appear in contrast with previous functional findings; yet, it is uncertain to what extent functional and structural connectivity measures correlate in neonates as some studies show no correlations in networks involving the amygdala (Kanel et al., 2022) and there is spatially varying structural-functional connectivity coupling across the brain (Preti and Van De Ville, 2019). The Bayley-III results are in line with previous studies showing positive correlations between white matter FA or structural connectivity across the brain and cognition following preterm birth (e.g. (Ball et al., 2015; Barnett et al., 2018)). Future studies should determine if similar relationships are present in the term group.

The study has some limitations and several avenues for future research can be considered. First, our cohort is enriched for very preterm infants who are at risk of atypical development in broad domains that can include autistic traits, indicating that further work is required to understand whether findings generalise. Preterm infants are more likely to be diagnosed with autism (Laverty et al., 2021) and because our population is enriched for preterm birth, higher prevalence of autistic traits in the study group could be expected. Accordingly, we found higher Q-CHAT scores in preterm compared to the term group in our cohort as reported by others (Wong et al., 2014). However, only two preterm participants had scores > 39, a screening cut-off point shown to maximise sensitivity and specificity for autism diagnosis (Allison et al., 2021), and the Q-CHAT scores in our sample (mean = 27, range 13–41) appear lower than those recently reported in the term-dominant dHCP cohort (mean = 30.5, range 8–70) (Kleine et al., 2022). Q-CHAT instrument has also been shown to have poor positive predictive value for autism, thus, the scores may instead reflect developmental/behavioural differences (Allison et al., 2021, Kleine et al., 2022). Therefore, the extent to which these findings generalise to the prediction of future autism diagnosis remains to be tested in future studies.

Second, the mothers of 70% of our participants had university/postgraduate degrees, so the dataset is skewed towards higher socioeconomic status, which can impact early child development. Maternal education was included as a covariate, but further studies are needed to confirm if the findings generalise to the wider population. Third, our study primarily used parent-completed questionnaires for outcome measures, which, despite being at risk for reporting biases, are considered to have good ecological validity (Diamond and Squires, 1993). Future work should contextualise these results with complementary direct measures including diagnostic assessment tools.

## 5 Conclusions

In summary, our results suggest that variation in the microstructure of the neonatal amygdala correlates with the frequency of autistic traits in infants born across the gestational range, potentially offering new insights into the neural basis developmental conditions that include autistic traits in their phenomenology.

## CRediT authorship contribution statement

**David Q Stoye:** Writing – review & editing, Resources, Investigation, Data curation. **Amy Corrigan:** Writing – review & editing, Resources, Investigation, Data curation. **Rebekah Smikle:** Writing – review & editing, Investigation. **Helen L Turner:** Writing – review & editing, Investigation. **Samuel R Neal:** Writing – review & editing, Writing – original draft, Visualization, Software, Methodology, Formal analysis, Data curation, Conceptualization. **Kadi Vaher:** Writing – review & editing, Writing – original draft, Visualization, Supervision, Software, Methodology, Investigation, Formal analysis, Data curation, Conceptualization. **Lorena Jiménez-Sánchez:** Writing – review & editing, Resources, Investigation. **Manuel Blesa Cábez:** Writing – review & editing, Software, Methodology, Data curation. **James P Boardman:** Writing – review & editing, Supervision, Resources, Project administration, Funding acquisition, Conceptualization. **Rebecca M Reynolds:** Writing – review & editing, Resources, Methodology, Funding acquisition. **Magda Rudnicka:** Writing – review & editing, Investigation. **Hilary Cruickshank:** Writing – review & editing, Investigation. **Michael J Thrippleton:** Writing – review & editing, Software, Resources, Methodology. **Mark E Bastin:** Writing – review & editing, Software, Resources, Methodology.

## Declaration of Competing Interest

All authors declare that they have no known competing financial interests or personal relationships that could have appeared to influence the work reported in this paper

## Data Availability

Data access process and analysis code repository are detailed in section 2.6 Data and code availability.
