# Supplementary material for: Neonatal amygdala microstructure and structural connectivity are associated with autistic traits at 2 years of age
Source: Dev Cogn Neurosci. 2026 Apr 7;79:101721. doi: 10.1016/j.dcn.2026.101721 (PMC13091228; doi:10.1016/j.dcn.2026.101721)
Supplement: Supplementary file 1 — Supplementary material [file mmc1.docx]

**Supplementary information to *Neonatal amygdala microstructure and structural connectivity are associated with autistic traits at 2 years of age***

Kadi Vaher, Samuel R Neal, Manuel Blesa Cábez, Lorena Jiménez-Sánchez, Amy Corrigan, David Q Stoye, Helen L Turner, Rebekah Smikle, Hilary Cruickshank, Magda Rudnicka, Mark E Bastin, Michael J Thrippleton, Rebecca M Reynolds, James P Boardman

# supplementary methods

## Image acquisition

Full imaging protocol is available in the study protocol paper (Boardman et al., 2020). Briefly, a 16-channel phased-array paediatric head coil was used to acquire 3D T2-weighted SPACE images (T2w) (voxel size = 1 mm isotropic, TE = 409 ms and TR = 3200 ms; acquisition time = 2:13 min) and axial diffusion MRI (dMRI) data. dMRI images were acquired in two separate acquisitions to reduce the time needed to re-acquire any data lost to motion artifacts: the first acquisition consisted of 8 baseline volumes (b = 0 s/mm^2^ [b0]) and 64 volumes with b = 750 s/mm^2^; the second consisted of 8 b0, 3 volumes with b = 200 s/mm^2^, 6 volumes with b = 500 s/mm^2^ and 64 volumes with b = 2500 s/mm^2^ (acquisition time = 4:29 + 5:01 min). An optimal angular coverage for the sampling scheme was applied (Caruyer et al., 2013). An acquisition of 3 b0 volumes with an inverse phase encoding direction was also performed (acquisition time = 0:28 min). All dMRI images were acquired using single-shot spin-echo echo planar imaging (EPI) with 2-fold simultaneous multislice and 2-fold in-plane parallel imaging acceleration and 2 mm isotropic voxels; all three diffusion acquisitions had the same parameters (TR/TE 3400/78.0 ms).

Infants were fed and wrapped and allowed to sleep naturally in the scanner. Pulse oximetry, electrocardiography and temperature were monitored. Flexible earplugs and neonatal earmuffs (MiniMuffs, Natus) were used for acoustic protection. All scans were supervised by a doctor or nurse trained in neonatal resuscitation. Each acquisition was inspected contemporaneously for motion artefact and repeated if there had been movement but the baby was still sleeping; dMRI acquisitions were repeated if signal loss was seen in 3 or more volumes.

## Covariate selection and inclusion

Based on literature, we considered the following potential confounders given their relationships with neonatal brain structure and/or neurodevelopment: GA at birth (Gale-Grant et al., 2022; Thompson et al., 2019b), birthweight and birthweight z-score (Thompson et al., 2019a), sex (Barnett et al., 2018; Hay et al., 2020; Nolvi et al., 2021; Thompson et al., 2019a), breast milk feeding (Belfort et al., 2016; Blesa et al., 2019), infant ethnicity (Freeman Duncan et al., 2012), maternal age (Du et al., 2021), maternal BMI at pregnancy booking (Oken et al., 2021; Salzwedel et al., 2019), maternal smoking during pregnancy (Ekblad et al., 2015), maternal postnatal depression (Kleine et al., 2022), and maternal highest educational qualification (Mckinnon et al., 2023; Sentenac et al., 2022). Coding and type of these variables are detailed in Supplementary Table 1. We used Pearson correlation to investigate correlations between continuous variables and neurodevelopmental outcomes, and two-sample t-tests to compare outcomes between groups defined by categorical nominal variables. Variables that were nominally significantly (p < 0.05) associated with at least one outcome measure were controlled for in downstream statistical analyses. These were GA at birth, sex, ethnicity, maternal postnatal depression score (dichotomised as ≤10), maternal age and maternal education. We additionally adjusted for GA at scan due to its strong correlation with brain MRI metrics.

## Maternal hair cortisol concentration

Sampling and measurement of maternal hair cortisol concentration is detailed in our previous publication (Stoye et al., 2020). Briefly, maternal hair was sampled within 10 days of delivery. Hair was cut close to the scalp, at the posterior vertex, and stored in aluminium foil at −20°C. The proximal 3 cm of hair were analysed by liquid chromatography-tandem mass spectrometry (LC-MS/MS), at Dresden Lab Service GmbH (Dresden, Germany), using an established protocol (Gao et al., 2013).

# supplementary tables

Please note that Tables S2-5 are provided in a separate Excel document.

Table S1. Coding and type of potential covariates collected through questionnaires and medical records.

| **Variable** | **Coded as** | **Type** |
| --- | --- | --- |
| Infant sex | Male or female | Categorical nominal |
| Infant gestational age at birth | Weeks | Continuous |
| Infant birthweight | Grams | Continuous |
| Birthweight z-score | Weight z-score calculated based on the International Fetal and Newborn Growth Consortium for the 21st Century (INTERGROWTH-21st) standards for preterm infants (Villar et al., 2016) | Continuous |
| Infant gestational age at MRI scan | Weeks | Continuous |
| Infant ethnicity | Any white background or any other ethnic group | Categorical nominal |
| Infant feeding at discharge | Exclusive breastmilk/mixed feeding or exclusive formula feeding | Categorical nominal |
| Maternal age | Years | Continuous |
| Maternal BMI at pregnancy booking | kg/m2 | Continuous |
| Maternal education (i.e. mother’s final educational qualification) | Data was obtained as following:   - 1 = none - 2 = 1-4 National 5s / Standard Grades / General Certificate of Secondary Education - 3 = > 5 National 5s / Standard Grades / General Certificate of Secondary Education - 4 = A levels / Highers / equivalent - 5 = College qualification (e.g. National Certificate, Higher National Certificate, Higher National Diploma) - 6 = University undergraduate degree - 7 = University postgraduate degree   From this data we created a dichotomous variable by combining brackets 1-5 and 6-7 to indicate whether the mother had obtained a university/postgraduate degree. | Categorical nominal |
| Maternal risk for postnatal depression | Score of 10 or higher on the self-reported Edinburgh Postnatal Depression Scale (Cox et al., 1987) at the MRI appointment at term-equivalent age. | Categorical nominal |
| Maternal smoking | Current smoker or never/ex-smoker | Categorical nominal |

# Supplementary figures


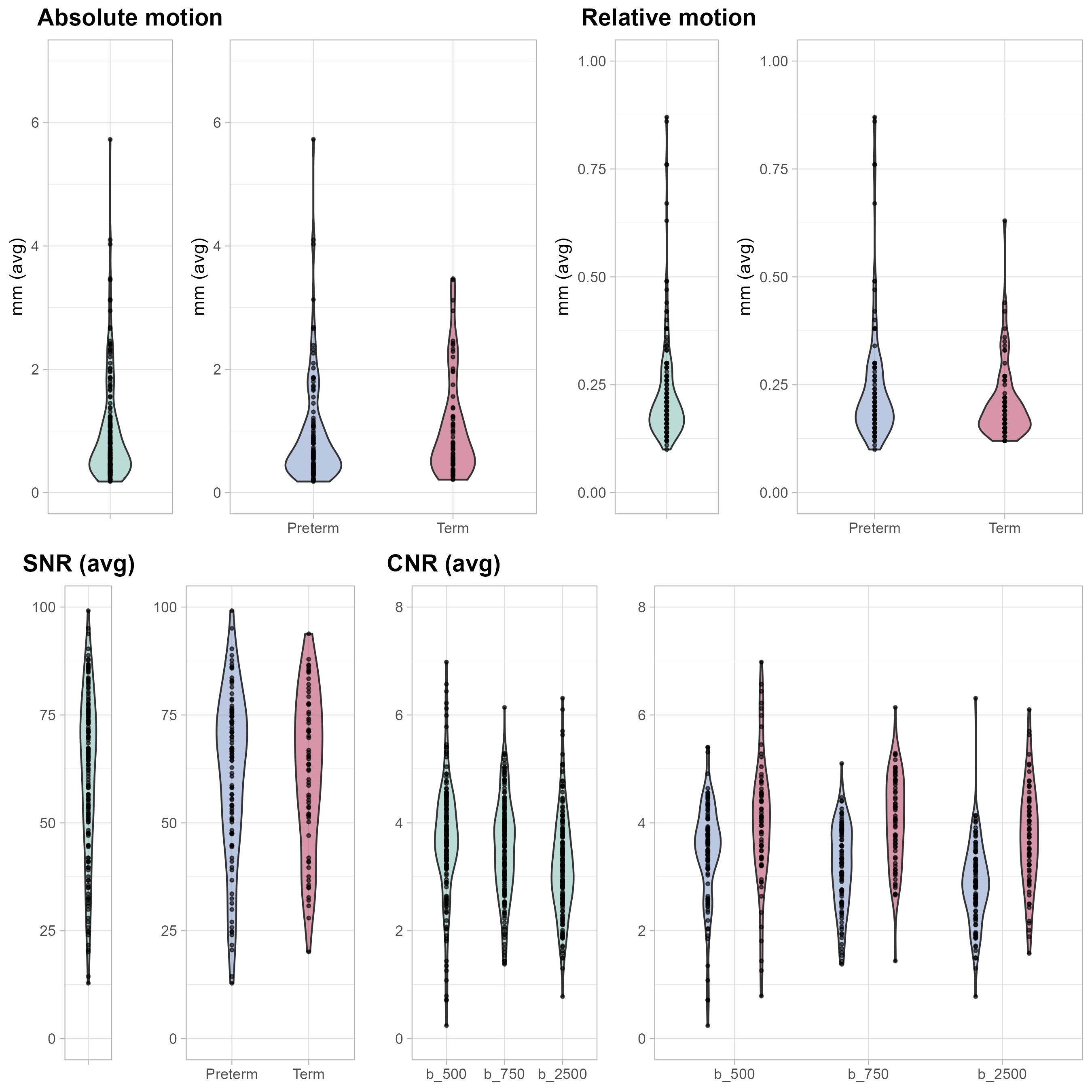


Figure S1. Eddy QC results. The right panels (green) show the distributions of the quality control metrics for the overall cohort, while the right panels show the distributions of the metrics for preterm (blue) and term (pink) infants separately. SNR = signal-to-noise ratio, CNR = contrast-to-noise ratio.


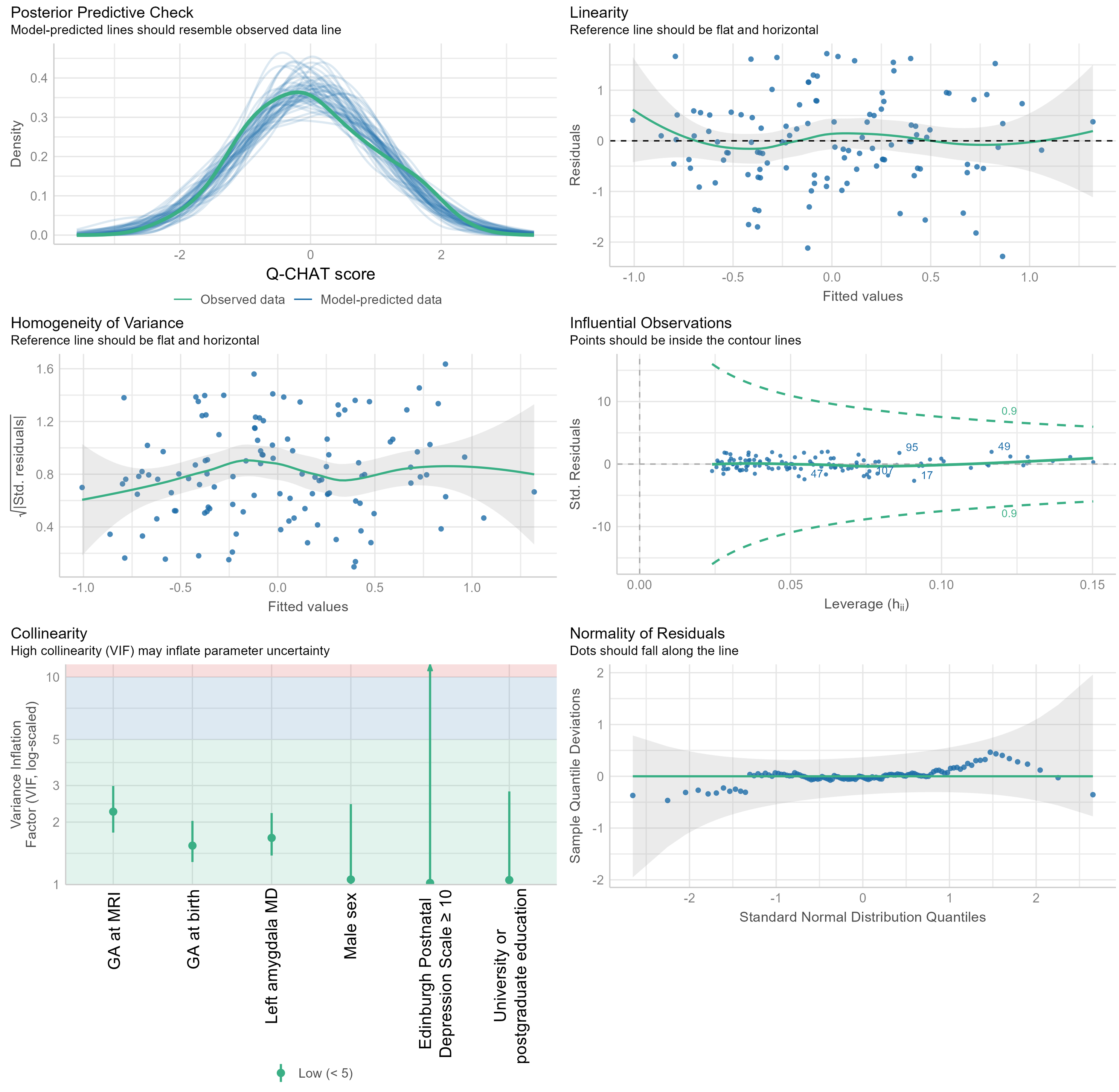


Figure S2. An example of model diagnostic plots shown for the regression model testing for the associations between left amygdala mean diffusivity and Q-CHAT.


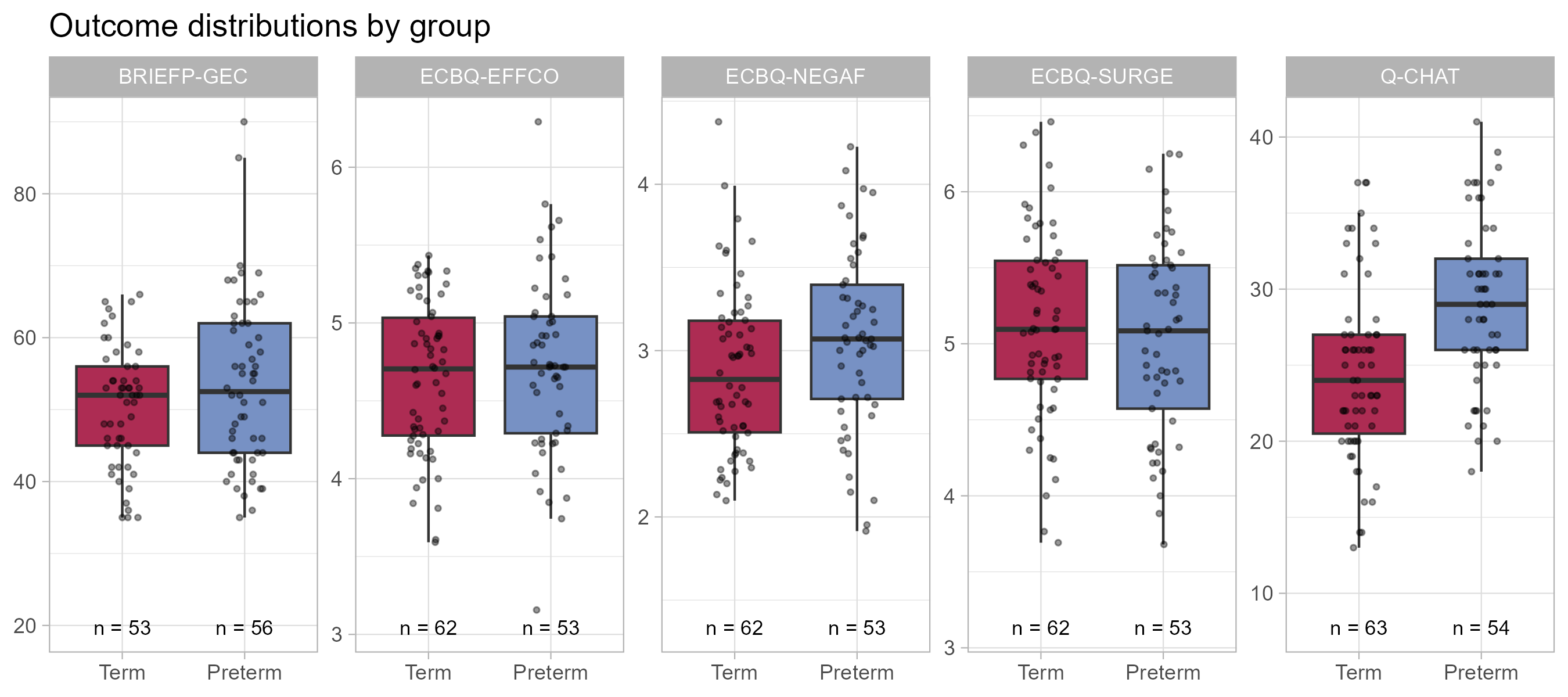


Figure S3. Distribution of 2-year outcomes in term and preterm groups. Welch t-test indicated a higher Q-CHAT score (t = -4.345, p = 2.99 × 10^-5^) and a slightly higher ECBQ negative affectivity trait in preterm compared to the term group (t = -1.901, p = 0.060). BRIEF-P = Behavior Rating Inventory of Executive Function, Preschool, GEC = global executive composite, Q-CHAT = Quantitative Checklist for Autism in Toddlers; ECBQ = Early Childhood Behavior Questionnaire; EFFCO = effortful control, NEGAF = negative affectivity, SURGE = surgency.


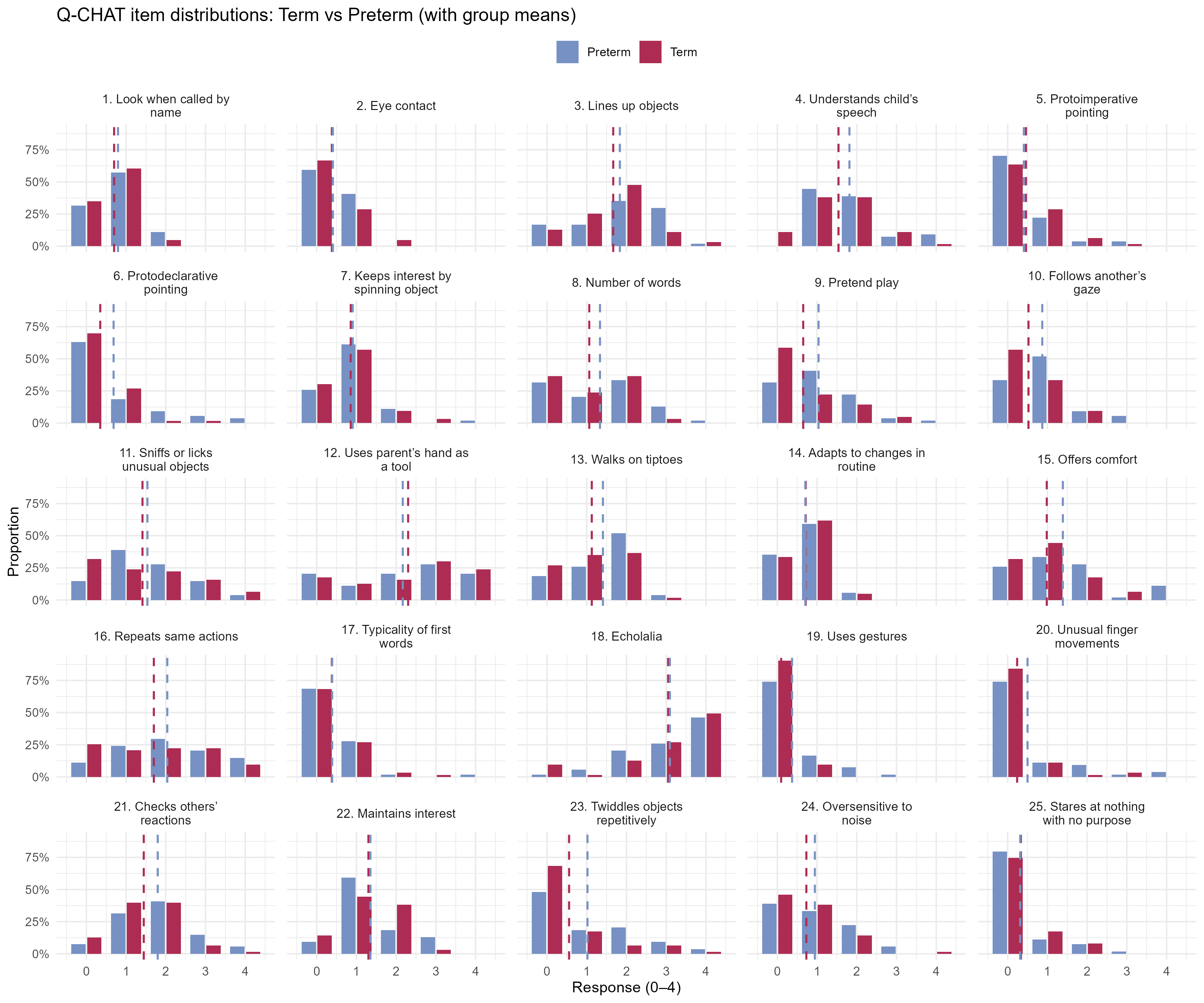


Figure S4. Distribution of Q-CHAT individual item responses in term and preterm groups. Dashed lines indicate group means per item.


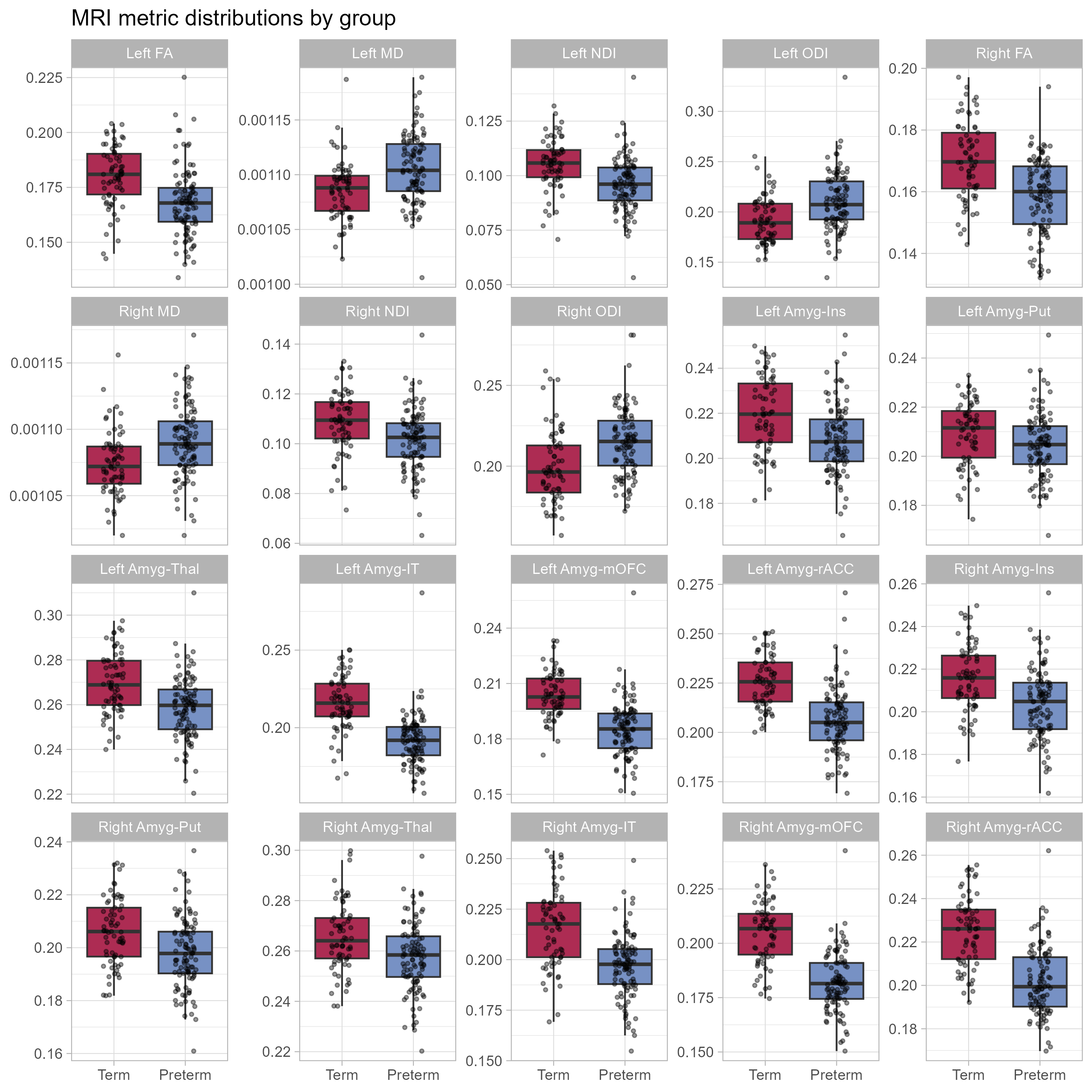


Figure S5. Distribution of neonatal amygdala imaging metrics in term and preterm infants. Please see Table S2 for statistical comparisons. FA = fractional anisotropy; MD = mean diffusivity; NDI = neurite density index; ODI = orientation dispersion index; Amyg = amygdala; Ins = insula; Put = putamen; Thal = thalamus; IT = inferior temporal gyrus; mOFC = medial orbitofrontal cortex; rACC = rostral anterior cingulate.


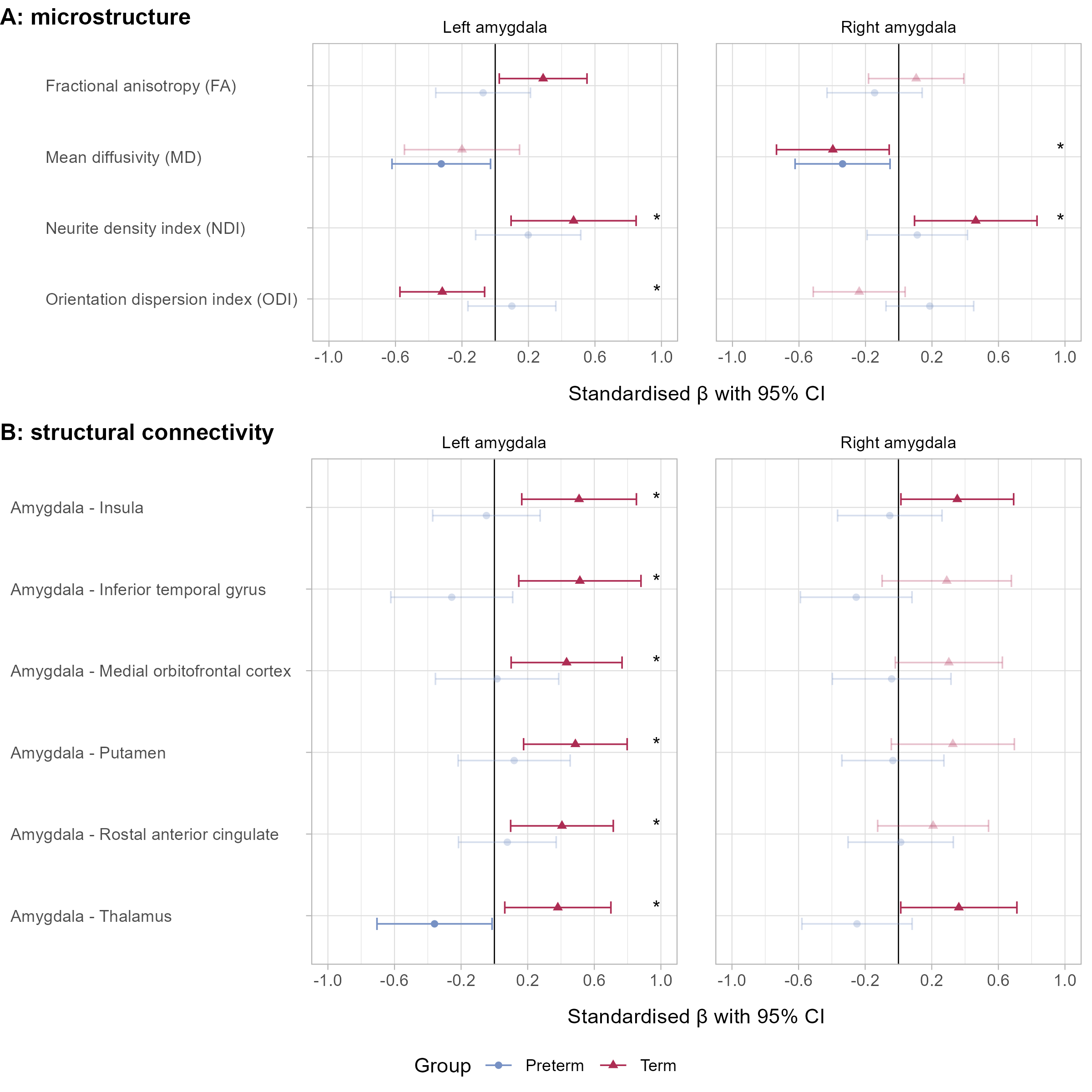


Figure S6. Amygdala microstructure (A) and structural connectivity (B) associations with Q-CHAT scores in term (blue lines and triangular shapes) and preterm groups (red lines and circular shapes). Models are adjusted for gestational age at birth and at scan, infant sex, maternal education (university/postgraduate degree vs lower) and high postnatal depression screening score (Edinburgh Postnatal Depression Scale ≥ 10). Lines and shapes shown as full colours indicate nominally statistically significant associations (p < 0.05), asterisks (*) indicate statistically significant associations after adjustment for multiple comparisons using the Benjamini-Hochberg procedure applied separately within the term and preterm groups.


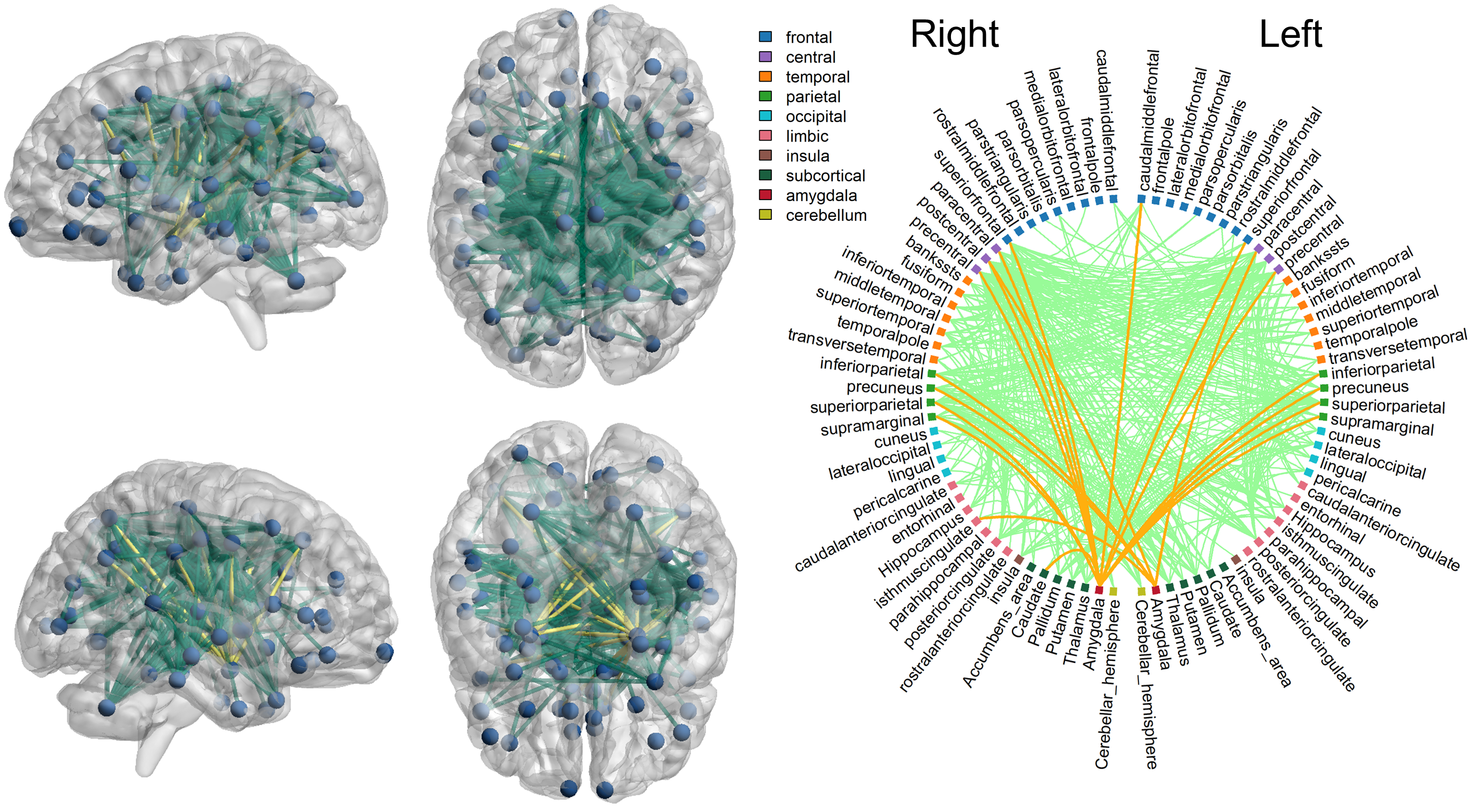


Figure S7. Illustration of network-based statistics results for ECBQ surgency dimension in preterm infants. Connections that appear as significant in at least 33.3% of experiments (mostly at test-statistic thresholds from 1.5 to 2.2) are shown; amygdala connections are shown in yellow/orange. The connections are between left amygdala and left paracentral gyrus, right inferior parietal cortex, right isthmus cingulate, right postcentral gyrus and right precuneus, and between right amygdala and left caudal middle frontal cortex, left inferior parietal cortex, left precentral gyrus, left precuneus, left superior frontal cortex, left superior parietal cortex, left supramarginal gyrus, right caudate, right paracentral gyrus, right postcentral gyrus, right precentral gyrus, right superior frontal cortex, right superior parietal cortex, and right supramarginal gyrus. All connections within the visualised network were positively associated with ECBQ surgency score.


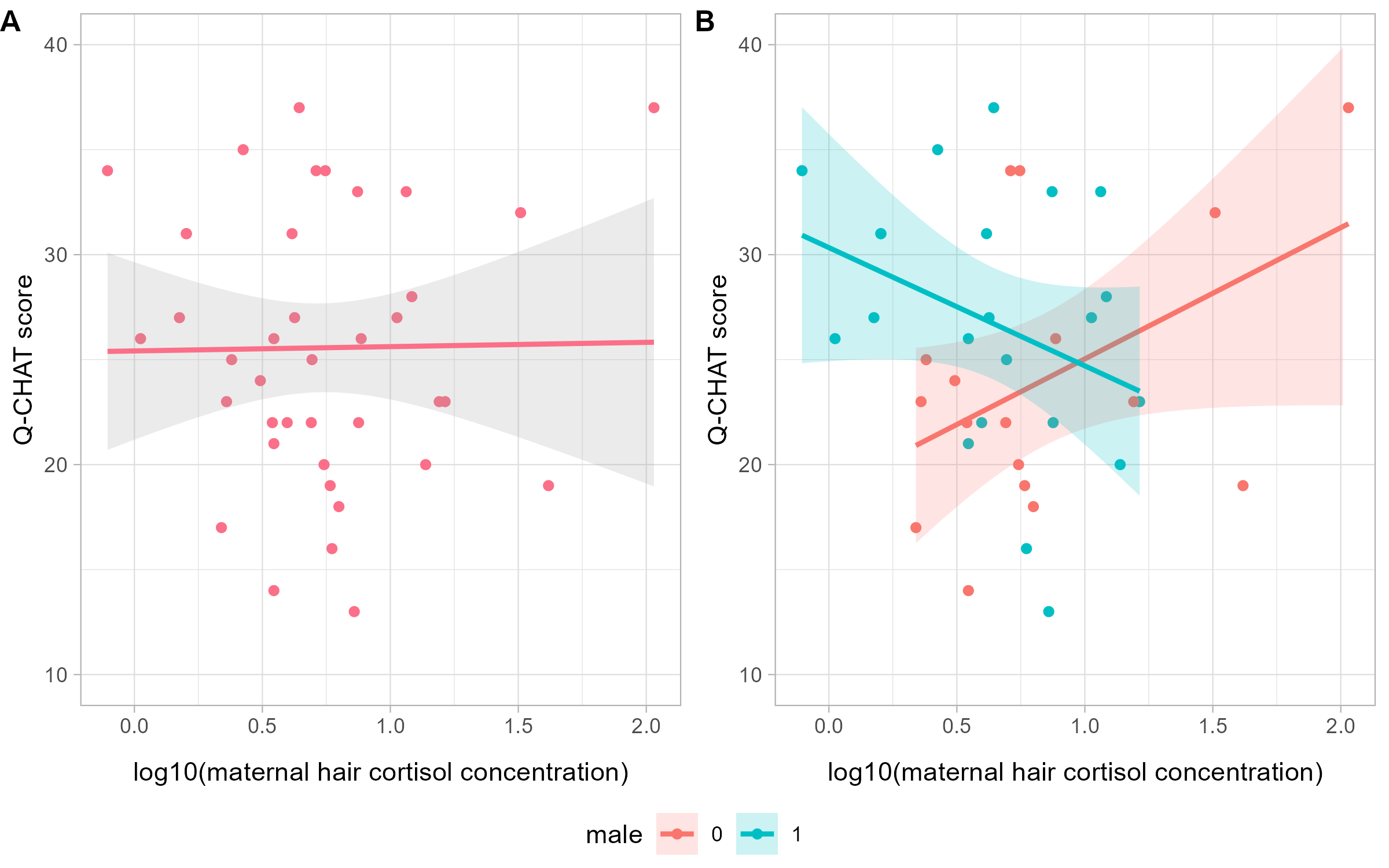


Figure S8. Maternal hair cortisol and Q-CHAT. (A) There is no correlation between Q-CHAT score and maternal hair cortisol concentration across the whole subsample (n=39). (B) Sex-specific correlations between maternal hair cortisol concentration and Q-CHAT (β_interaction_ =-0.661, p=0.043, adjusted for GA at birth, n=39).

# supplementary references

Barnett, M.L., Tusor, N., Ball, G., Chew, A., Falconer, S., Aljabar, P., Kimpton, J.A., Kennea, N., Rutherford, M., David Edwards, A., Counsell, S.J., 2018. Exploring the multiple-hit hypothesis of preterm white matter damage using diffusion MRI. NeuroImage Clin. 17, 596–606. https://doi.org/10.1016/j.nicl.2017.11.017

Belfort, M.B., Anderson, P.J., Nowak, V.A., Lee, K.J., Molesworth, C., Thompson, D.K., Doyle, L.W., Inder, T.E., 2016. Breast Milk Feeding, Brain Development, and Neurocognitive Outcomes: A 7-Year Longitudinal Study in Infants Born at Less Than 30 Weeks’ Gestation. J. Pediatr. 177, 133–139. https://doi.org/10.1016/j.jpeds.2016.06.045

Blesa, M., Sullivan, G., Anblagan, D., Telford, E.J., Quigley, A.J., Sparrow, S.A., Serag, A., Semple, S.I., Bastin, M.E., Boardman, J.P., 2019. Early breast milk exposure modifies brain connectivity in preterm infants. Neuroimage 184, 431–439. https://doi.org/10.1016/J.NEUROIMAGE.2018.09.045

Boardman, J.P., Hall, J., Thrippleton, M.J., Reynolds, R.M., Bogaert, D., Davidson, D.J., Schwarze, J., Drake, A.J., Chandran, S., Bastin, M.E., Fletcher-Watson, S., 2020. Impact of preterm birth on brain development and long-term outcome: protocol for a cohort study in Scotland. BMJ Open 10, 35854. https://doi.org/10.1136/bmjopen-2019-035854

Caruyer, E., Lenglet, C., Sapiro, G., Deriche, R., 2013. Design of multishell sampling schemes with uniform coverage in diffusion MRI. Magn. Reson. Med. 69, 1534–1540. https://doi.org/10.1002/mrm.24736

Cox, J.L., Holden, J.M., Sagovsky, R., 1987. Detection of Postnatal Depression: Development of the 10-item Edinburgh Postnatal Depression scale. Br. J. Psychiatry 150, 782–786. https://doi.org/10.1192/bjp.150.6.782

Du, J., Rolls, E.T., Gong, W., Cao, M., Vatansever, D., Zhang, J., Kang, J., Cheng, W., Feng, J., 2021. Association between parental age, brain structure, and behavioral and cognitive problems in children. Mol. Psychiatry 27, 967–975. https://doi.org/10.1038/s41380-021-01325-5

Ekblad, M., Korkeila, J., Lehtonen, L., 2015. Smoking during pregnancy affects foetal brain development. Acta Paediatr. 104, 12–18. https://doi.org/10.1111/APA.12791

Freeman Duncan, A., Watterberg, K.L., Nolen, T.L., Vohr, B.R., Adams-Chapman, I., Das, A., Lowe, J., 2012. Effect of Ethnicity and Race on Cognitive and Language Testing at Age 18-22 Months in Extremely Preterm Infants. J. Pediatr. 160, 966-971.e2. https://doi.org/10.1016/J.JPEDS.2011.12.009

Gale-Grant, O., Fenn-Moltu, S., França, L.G.S., Dimitrova, R., Christiaens, D., Cordero-Grande, L., Chew, A., Falconer, S., Harper, N., Price, A.N., Hutter, J., Hughes, E., O’Muircheartaigh, J., Rutherford, M., Counsell, S.J., Rueckert, D., Nosarti, C., Hajnal, J. V., McAlonan, G., Arichi, T., Edwards, A.D., Batalle, D., 2022. Effects of gestational age at birth on perinatal structural brain development in healthy term-born babies. Hum. Brain Mapp. 43, 1577–1589. https://doi.org/10.1002/hbm.25743

Gao, W., Stalder, T., Foley, P., Rauh, M., Deng, H., Kirschbaum, C., 2013. Quantitative analysis of steroid hormones in human hair using a column-switching LC-APCI-MS/MS assay. J. Chromatogr. B Anal. Technol. Biomed. Life Sci. 928, 1–8. https://doi.org/10.1016/j.jchromb.2013.03.008

Hay, R.E., Reynolds, J.E., Grohs, M.N., Paniukov, D., Giesbrecht, G.F., Letourneau, N., Dewey, D., Lebel, C., 2020. Amygdala-Prefrontal Structural Connectivity Mediates the Relationship between Prenatal Depression and Behavior in Preschool Boys. J. Neurosci. 40, 6969–6977. https://doi.org/10.1523/JNEUROSCI.0481-20.2020

Kleine, I., Vamvakas, G., Lautarescu, A., Falconer, S., Chew, A., Counsell, S., Pickles, A., Edwards, D., Nosarti, C., 2022. Postnatal maternal depressive symptoms and behavioural outcomes in term-born and preterm-born toddlers: a longitudinal UK community cohort study. BMJ Open 12, e058540. https://doi.org/10.1136/BMJOPEN-2021-058540

Mckinnon, K., Galdi, P., Blesa-Cábez, M., Sullivan, G., Vaher, K., Corrigan, A., Hall, J., Jiménez-Sánchez, L., Thrippleton, M., Bastin, M.E., Quigley, A.J., Valavani, E., Tsanas, A., Richardson, H., Boardman, J.P., 2023. Association of Preterm Birth and Socioeconomic Status With Neonatal Brain Structure. JAMA Netw. Open 6, e2316067–e2316067. https://doi.org/10.1001/JAMANETWORKOPEN.2023.16067

Nolvi, S., Tuulari, J.J., Pelto, J., Bridgett, D.J., Eskola, E., Lehtola, S.J., Hashempour, N., Korja, R., Kataja, E.L., Saunavaara, J., Parkkola, R., Lähdesmäki, T., Scheinin, N.M., Fernandes, M., Karlsson, L., Lewis, J.D., Fonov, V.S., Collins, D.L., Karlsson, H., 2021. Neonatal amygdala volumes and the development of self-regulation from early infancy to toddlerhood. Neuropsychology 35, 285–299. https://doi.org/10.1037/NEU0000724

Oken, E., Thompson, J.W., Rifas-Shiman, S.L., Vilchuk, K., Bogdanovich, N., Hameza, M., Yang, S., Patel, R., Kramer, M.S., Martin, R.M., 2021. Analysis of Maternal Prenatal Weight and Offspring Cognition and Behavior: Results From the Promotion of Breastfeeding Intervention Trial (PROBIT) Cohort. JAMA Netw. Open 4, e2121429–e2121429. https://doi.org/10.1001/JAMANETWORKOPEN.2021.21429

Salzwedel, A.P., Gao, W., Andres, A., Badger, T.M., Glasier, C.M., Ramakrishnaiah, R.H., Rowell, A.C., Ou, X., 2019. Maternal Adiposity Influences Neonatal Brain Functional Connectivity. Front. Hum. Neurosci. 12. https://doi.org/10.3389/FNHUM.2018.00514

Sentenac, M., Benhammou, V., Aden, U., Ancel, P.Y., Bakker, L.A., Bakoy, H., Barros, H., Baumann, N., Bilsteen, J.F., Boerch, K., Croci, I., Cuttini, M., Draper, E., Halvorsen, T., Johnson, S., Källén, K., Land, T., Lebeer, J., Lehtonen, L., Maier, R.F., Marlow, N., Morgan, A., Ni, Y., Raikkonen, K., Rtimi, A., Sarrechia, I., Varendi, H., Vollsaeter, M., Wolke, D., Ylijoki, M., Zeitlin, J., 2022. Maternal education and cognitive development in 15 European very-preterm birth cohorts from the RECAP Preterm platform. Int. J. Epidemiol. 50, 1824–1839. https://doi.org/10.1093/IJE/DYAB170

Stoye, D.Q., Blesa, M., Sullivan, G., Galdi, P., Lamb, G.J., Black, G.S., Quigley, A.J., Thrippleton, M.J., Bastin, M.E., Reynolds, R.M., Boardman, J.P., 2020. Maternal cortisol is associated with neonatal amygdala microstructure and connectivity in a sexually dimorphic manner. Elife 9, e60729. https://doi.org/10.7554/ELIFE.60729

Thompson, D.K., Kelly, C.E., Chen, J., Beare, R., Alexander, B., Seal, M.L., Lee, K., Matthews, L.G., Anderson, P.J., Doyle, L.W., Spittle, A.J., Cheong, J.L.Y., 2019a. Early life predictors of brain development at term-equivalent age in infants born across the gestational age spectrum. Neuroimage 185, 813–824. https://doi.org/10.1016/J.NEUROIMAGE.2018.04.031

Thompson, D.K., Kelly, C.E., Chen, J., Beare, R., Alexander, B., Seal, M.L., Lee, K.J., Matthews, L.G., Anderson, P.J., Doyle, L.W., Cheong, J.L.Y., Spittle, A.J., 2019b. Characterisation of brain volume and microstructure at term-equivalent age in infants born across the gestational age spectrum. NeuroImage Clin. 21, 101630. https://doi.org/10.1016/j.nicl.2018.101630

Villar, J., Giuliani, F., Fenton, T.R., Ohuma, E.O., Ismail, L.C., Kennedy, S.H., 2016. INTERGROWTH-21st very preterm size at birth reference charts. Lancet 387, 844–845. https://doi.org/10.1016/S0140-6736(16)00384-6
